# A Broad Spectrum Chemokine Inhibitor Prevents Preterm Labor but Not Microbial Invasion of the Amniotic Cavity or Neonatal Morbidity in a Non-human Primate Model

**DOI:** 10.3389/fimmu.2020.00770

**Published:** 2020-04-30

**Authors:** Michelle Coleman, Austyn Orvis, Tsung-Yen Wu, Matthew Dacanay, Sean Merillat, Jason Ogle, Audrey Baldessari, Nicole M. Kretzer, Jeff Munson, Adam J. Boros-Rausch, Oksana Shynlova, Stephen Lye, Lakshmi Rajagopal, Kristina M. Adams Waldorf

**Affiliations:** ^1^Center for Global Infectious Disease Research, Seattle Children's Research Institute, Seattle, WA, United States; ^2^Department of Obstetrics & Gynecology, University of Washington, Seattle, WA, United States; ^3^Washington National Primate Center, University of Washington, Seattle, WA, United States; ^4^Department of Psychiatry and Behavioral Sciences, University of Washington, Seattle, WA, United States; ^5^Department of Physiology, University of Toronto, Toronto, ON, Canada; ^6^Department of Obstetrics & Gynaecology, University of Toronto, Toronto, ON, Canada; ^7^Department of Pediatrics, University of Washington, Seattle, WA, United States; ^8^Department of Global Health, University of Washington, Seattle, WA, United States

**Keywords:** chorioamniotic membranes, placenta, neutrophil, chemokine, infection, fetus, preterm labor, group B streptoccocus

## Abstract

Leukocyte activation within the chorioamniotic membranes is strongly associated with inflammation and preterm labor (PTL). We hypothesized that prophylaxis with a broad-spectrum chemokine inhibitor (BSCI) would downregulate the inflammatory microenvironment induced by Group B Streptococcus (GBS, *Streptococcus agalactiae*) to suppress PTL and microbial invasion of the amniotic cavity (MIAC). To correlate BSCI administration with PTL and MIAC, we used a unique chronically catheterized non-human primate model of Group B Streptococcus (GBS)-induced PTL. In the early third trimester (128–138 days gestation; ~29–32 weeks human pregnancy), animals received choriodecidual inoculations of either: (1) saline (*N* = 6), (2) GBS, 1–5 × 10^8^ colony forming units (CFU)/ml; *N* = 5), or (3) pre-treatment and daily infusions of a BSCI (10 mg/kg intravenous and intra-amniotic) with GBS (1–5 × 10^8^ CFU/ml; *N* = 4). We measured amniotic cavity pressure (uterine contraction strength) and sampled amniotic fluid (AF) and maternal blood serially and cord blood at delivery. Cesarean section was performed 3 days post-inoculation or earlier for PTL. Data analysis used Fisher's exact test, Wilcoxon rank sum and one-way ANOVA with Bonferroni correction. Saline inoculation did not induce PTL or infectious sequelae. In contrast, GBS inoculation typically induced PTL (4/5, 80%), MIAC and fetal bacteremia (3/5; 60%). Remarkably, PTL did not occur in the BSCI+GBS group (0/4, 0%; *p* = 0.02 vs. GBS), despite MIAC and fetal bacteremia in all cases (4/4; 100%). Compared to the GBS group, BSCI prophylaxis was associated with significantly lower cytokine levels including lower IL-8 in amniotic fluid (*p* = 0.03), TNF-α in fetal plasma (*p* < 0.05), IFN-α and IL-7 in the fetal lung (*p* = 0.02) and IL-18, IL-2, and IL-7 in the fetal brain (*p* = 0.03). Neutrophilic chorioamnionitis was common in the BSCI and GBS groups, but was more severe in the BSCI+GBS group with greater myeloperoxidase staining (granulocyte marker) in the amnion and chorion (*p* < 0.05 vs. GBS). Collectively, these observations indicate that blocking the chemokine response to infection powerfully suppressed uterine contractility, PTL and the cytokine response, but did not prevent MIAC and fetal pneumonia. Development of PTL immunotherapies should occur in tandem with evaluation for AF microbes and consideration for antibiotic therapy.

## Introduction

Preterm birth is the single most important cause of perinatal mortality and morbidity and a major contributor to perinatal and infant mortality worldwide. Annually, there are more than 15 million preterm births, which contribute to more than 1 million deaths globally (1, 2). The neonatal sequelae of preterm birth can be significant and lead to long-term disability, cognitive impairment, blindness, and pulmonary complications (3–6). There is a differential impact upon the small subset of early preterm births before 32 weeks gestation (1–2% of all births, 16% of preterm births), because these infants account for the majority of neurological morbidity and perinatal mortality (7, 8). Although progesterone supplementation was shown to prevent preterm birth in women with a history of a prior preterm birth, more recent trials have not shown benefit (9–13). Clearly, new approaches are urgently needed to prevent perinatal mortality and serious morbidities associated with preterm birth.

Mechanisms leading to the onset of labor involve a complex series of fetal and placental endocrine events that act to prime the myometrium. In a healthy and normal pregnancy, the uterus transitions from a quiescent organ to one that is able to efficiently propagate electrical signals to generate contractions near term (14, 15). Both term and preterm labor (PTL) is known to be an inflammatory process with immune cells and inflammatory proteins (cytokines, chemokines) playing a key role in parturition (16–20). We and others have demonstrated that inflammation within the myometrium and decidua *precedes* the onset of spontaneous and PTL, thereby implicating an inflammatory process as a labor inciting event (19, 21–26). Choriodecidual tissues at the maternal-fetal interface represent a primary site for the synchronized infiltration of peripheral leukocytes (21, 27, 28) that could have a direct effect on the myometrium (24, 27, 29) to promote uterine contractions and cervical ripening (16, 29–31). A pharmacologic block of inflammation within the myometrium, decidua and placenta may represent a useful therapeutic approach for preventing preterm birth.

Recruitment of leukocytes from the peripheral circulation to the decidua and myometrium is mediated by chemokines, a class of cytokines that act as chemoattractants (32, 33). Chemokines include ~50 endogenous chemokine ligands and 20 G protein-coupled receptors [reviewed in (32)]. In women with PTL, several chemokines are elevated in the amniotic fluid, placenta, decidua and/or myometrium including monocyte chemotactic protein 1 (MCP-1/CCL-2), chemokine (C-X-C motif) ligand 1 (CXCL1), interleukin-8 (IL-8/CXCL8), interleukin-6 (IL-6), and macrophage migration inhibitory factor (MIF) (28, 34–41). Chemokine receptor antagonists might inhibit PTL and have been used in clinical trials to prevent cancer metastasis (42, 43) and as an early stage HIV therapy (44). In rodent models, chemokine receptor antagonists have been used to prevent or ameliorate kidney disease (45–47), bowel inflammation (48, 49), and brain injury or stroke (50). Broad Spectrum Chemokine Inhibitors (BSCI) have also been developed that can simultaneously block multiple chemokine signaling pathways (51).

In this study, we used a BSCI, which specifically binds the cell-surface type-2 somatostatin receptor (SSTR2) and results in a potent suppression of chemokine signaling without directly affecting chemokine receptors (52–54). Our previous work showed that pre-treatment with the BSCI (BN83470) resulted in reduced uterine inflammation and partially prevented preterm birth induced by lipopolysaccharide (LPS) in a mouse model of preterm labor (55). The efficacy of a BSCI to ameliorate disease has been demonstrated in a wide range of animal models (e.g., allergic asthma, surgical adhesion formation, rheumatoid arthritis, and HIV replication) (51, 53, 56–60). This data provided the basis for this study that uses a new BSCI compound (FX125L) with superior pharmaceutical properties including pharmacokinetics, safety and toxicology with the potential for greater therapeutic efficacy [(61) and Dr. David Fox, Warwick University, personal communication]. Whether a BSCI, like FX125L, might prevent PTL by limiting leukocyte recruitment and inflammatory cascades within the chorioamniotic membranes and myometrium is unknown.

We hypothesized that prophylaxis with a BSCI would downregulate the inflammatory microenvironment induced by Group B Streptococcus (GBS, *Streptococcus agalactiae*) to suppress PTL, but still allow for bacterial resolution. GBS is a common lower genital tract bacteria associated with fetal injury, stillbirth, and preterm birth (62–71). To test our hypothesis, we used a unique chronically catheterized pregnant non-human primate (NHP; *Macaca nemestrina*, pigtail macaque) in which we have previously studied the pathogenesis of GBS-induced inflammation and preterm birth (72–76). In this model, we inoculate GBS into the choriodecidual space, between the uterine muscle and the chorioamniotic membranes, where bacteria are first thought to come in contact with the choriodecidual tissues and myometrium after ascending from the vagina (72, 75, 77). Herein, we demonstrate that prophylactic BSCI administration reduced the rate of PTL, but failed to limit bacterial invasion of the amniotic cavity and fetus, which led to greater fetal inflammation and injury.

## Materials and Methods

### Ethics Statement

All animal experiments were carried out in strict accordance with the recommendations in the Guide for the Care and Use of Laboratory Animals of the National Research Council and the Weather all report, “The use of non-human primates in research.” The University of Washington Institutional Animal Care Use Committee approved the protocol (Permit Number: 4165-01). All surgery was performed under general anesthesia and all efforts were made to minimize suffering.

Written informed consent for donation of adult human blood was obtained from subjects, per the Principles in the WMA Declaration of Helsinki and Dept. of Health and Human Services Belmont Report. The study was approved by the Seattle Children's Research Institute Institutional Review Board (protocol #11117). Children under the age of 18 were not recruited for donation of human blood.

### Study Design and the Chronically Catheterized NHP Model

We used a chronically catheterized pregnant NHP (*Macaca nemestrina*) model, in which we surgically implanted catheters via laparotomy into the maternal femoral vein, amniotic cavity, and choriodecidual interface in the lower uterine segment (between uterine muscle and fetal membranes, external to amniotic fluid). For inducing PTL, we used a hyperhemolytic and hyperpigmented GBS strain (GBS COH1Δ*covR*), lacking the hemolysin repressor CovR, which is associated with PTL (63, 75). In this study, animals received choriodecidual infusions of GBS COH1Δ*covR* with pre-treatment and daily infusions of a BSCI (*N* = 4; 10 mg/kg intravenous and 10 mg/kg intra-amniotic). These results were compared to two other groups of animals receiving either a choriodecidual inoculation of GBS COH1Δ*covR* (*N* = 5; hypervirulent, hyperpigmented strain, 1–5 × 10^8^ CFU/ml) or saline (*N* = 6). The GBS COH1Δ*covR* (*N* = 5) and some of the saline control (*N* = 4) experiments were performed and published previously (19, 72, 73). Other saline control experiments (*N* = 2) were performed as part of this study.

Our chronically catheterized NHP model has been previously described (75). Briefly, between days 114–125 of pregnancy (term = 172 days), catheters were surgically implanted via laparotomy into the maternal femoral artery and vein, amniotic cavity, and choriodecidual interface in the lower uterine segment (between uterine muscle and fetal membranes, external to the amniotic cavity). After surgery, the animal was placed in the jacket and tether with catheters tracked through the tether system. Cefazolin, indomethacin and either terbutaline sulfate or atosiban were administered to reduce postoperative infection risk and uterine activity, but stopped at least 72 h before experimental start (~5 half-lives for terbutaline, 40 half-lives for cefazolin, >97% of both drugs eliminated). Experiments began ~2 weeks after catheterization surgery to allow recovery (~30–31 weeks human gestation). Intra-amniotic pressure was continuously recorded, digitized, and analyzed using custom software. The integrated area under the intrauterine pressure curve was used as a quantitative measure of uterine activity and reported as the hourly contraction area (HCA; mmHg-s/h) over 24 h.

### PTL and Cesarean Section

PTL was defined as progressive cervical dilation associated with increased uterine activity (>10,000 mmHg-s/h). Cesarean section was performed at the following endpoints to allow for tissue collection: (1) PTL, (2) 3 days after GBS inoculation if no PTL was observed, or (3) 7 days after saline inoculation (72). A 3-day endpoint to assess the effects of GBS on placental tissues and fetal injury was chosen in order to study the earliest events in the pathway of infection/inflammation associated preterm birth. The 7-day endpoint for saline controls was chosen at the inception of our research program and provides a close gestational age match for the current study. After Cesarean section, fetuses were euthanized by barbiturate overdose followed by exsanguination and fetal necropsy.

### BSCI

The BSCI (FX125L) was obtained as a gift from Dr. David Fox at the University of Warwick. A dose of 10 mg/kg, administered both intra-amniotically and intravenously, was chosen based on murine studies showing efficacy with an equivalent dose for the inhibition of LPS-induced PTL (55). The first infusion of BSCI was administered 24 h before GBS inoculation. The second infusion was given ~1 h prior to GBS inoculation. Subsequently, BSCI infusions were administered daily until Cesarean section.

### Bacterial Growth and Confirmation of GBS From Infected Animals

GBS strains used in this study were derived from a clinical isolate known as COH-1 (ST-17 clone, capsular serotype III), which was obtained from an infected newborn (78, 79). The hyperhemolytic and hyperpigmented Δ*covR* was derived from wild type (WT) GBS COH-1 (63) was previously tested in our NHP model (75). Routine cultures of GBS were grown in tryptic soy broth (TSB) or tryptic soy agar (TSA, Difco Laboratories) at 37°C in 5% CO_2_. For inoculations in the NHP model, GBS strains were grown to mid-log phase (O.D_600_ = 0.3) and ~1–5 × 10^8^ CFU in 1 mL PBS was inoculated into the choriodecidual space, as described previously (72). For bacterial enumeration, amniotic fluid (100 μL) was serially diluted and 10-fold dilutions were plated on TSA, incubated overnight at 37°C, 5% CO_2_ and enumerated to determine bacterial invasion. Quantities of GBS were reported as colony forming units (CFU).

To confirm that bacteria recovered from infected animals was GBS, we took advantage of the fact that the hyperpigmented GBSΔ*covR* strain exhibits an orange color on TSA (63). We also tested for CAMP factor activity on sheep blood agar plates with the inoculum strain included in parallel. PCR and DNA sequencing for 16S ribosomal RNA was performed as needed. These tests confirmed that GBS did not change phenotype during the course of the experiment.

### NHP Sample Collection

Amniotic fluid (AF) and maternal blood were sampled frequently i.e., before (−24 and −0.25 h) and after pathogen inoculation (+0.75, +6, +12, +24 h and then every 12 h until Cesarean section for fetal necropsy) to culture for GBS and assay for inflammatory mediators. At the time of Cesarean section, fetal blood was obtained. For cytokine and prostaglandin (PG) analysis, samples of amniotic fluid and blood were collected in heparin and EDTA tubes, respectively. Samples were centrifuged for 5 min at 1,200 rpm immediately after collection and the supernatant was frozen and stored at −80°C. For analysis of bacterial dissemination in fetal organs, fetal tissues were weighed at necropsy, homogenized in sterile PBS and 10-fold serial dilutions were plated on TSA and incubated overnight at 37°C, 5% CO_2_ and enumerated as described (80). For inflammatory cytokine analysis, fetal organ homogenates were diluted 1:1 in lysis buffer [150 mM NaCl, 15 mM Tris, 1 mM MgCl_2_, 1 mM CaCl_2_, 1% Triton X-100, supplemented with Complete Mini, EDTA-free protease inhibitor cocktail (Roche)] and incubated overnight at 4°C. Lysates were centrifuged at 4,000 rpm for 20 min at 4°C, and the supernatants were stored at −80°C or used immediately for analysis. About 100 μl of sample was used in Luminex or ELISA assays as described above.

### Cytokine and Prostaglandin Analysis

Cytokines [interleukin-1β (IL-1β), interleukin-6 (IL-6), interleukin (IL-8), and tumor necrosis factor-alpha (TNF-α)] levels were determined using Luminex multiplex cytokine kits (Millipore, Billerica, MA) for maternal plasma, fetal plasma and AF samples. Prostaglandin E2 (PGE2) and Prostaglandin F2-alpha (PGF2α) were determined using commercially available human EIA kits (Cayman Chemical, Ann Arbor, MI). In the tissues, we quantitated cytokines using a Luminex multiplex cytokine kit designed for NHP samples covering 37 cytokines/chemokines (37-plex ProcartaPlex™ NHP panel; Thermo Fisher Scientific, Waltham, MA). Values are reported as pg/mL in the amniotic fluid and maternal and fetal plasma or pg/g in fetal tissues.

### Histopathology, Immunohistochemistry and Quantitative Image Analysis

A board-certified veterinary pathologist (A.B) peformed histopathologic examination of the fetal and placental tissues. H&E-stained, full-thickness paraffin sections (placental disc, umbilical cord, fetal membrane roll) were examined from each case to exclude inflammation, necrosis, fetal vascular thrombosis, or other histopathological findings. Chorioamnionitis was diagnosed by the presence of a neutrophilic infiltrate at the chorion-decidua junction (mild) or amniochorion junction (moderate or severe). For histologic examination of the fetal lung, two to three randomly selected fixed fetal lung tissues were embedded in paraffin and sections stained with hematoxylin and eosin (H&E). We also performed immunostaining for myeloperoxidase (MPO, granulocyte marker) and CD68 (macrophage marker) on the chorioamniotic membranes and fetal lung tissues. For the chorioamniotic membrane analyses, we included an additional two controls (81).

The University of Washington Histology and Imaging Core performed the immunohistochemistry optimization and staining for MPO and CD68. We used the following primary, secondary and tertiary antibodies: rabbit polyclonal MPO (Clone Ab-1, ThermoScientific, Catalog Number RB-373-A1), mouse monoclonal CD68 (Clone 514H12, Leica, Catalog Number PA0273), Leica Post-Primary linker and goat anti-rabbit horseradish peroxidase polymerized antibody (Leica Catalog Number DS9800). Utilizing the Leica Bond Rx Automated Immunostainer (Leica Microsystems, Buffalo Grove, IL), slides were first deparaffinized with Leica Dewax Solution at 72°C for 30 s. For MPO staining, antigen retrieval was heat-mediated using citrate, pH 6, at 100°C for 20 min. For CD68 staining, antigen retrieval was performed with EDTA, pH 9, at 100°C for 20 min. All subsequent steps were at room temperature. Blocking was performed with 10% normal goat serum (Jackson ImmunoResearch, Catalog Number 005-000-121) in tris-buffered saline for 20 min followed by blocking with Leica Bond Peroxide Block for 5 min. Slides were then incubated with either the MPO (1:100) primary antibody in Leica Primary Antibody Diluent or CD68 (no dilution) primary antibody for 30 min. For MPO, a secondary antibody (goat anti-rabbit horseradish peroxidase polymerized antibody) was applied for 8 min. For CD68, the Leica Post-Primary linker was applied for 8 min and tissues were then incubated with a tertiary antibody (goat anti-rabbit horseradish peroxidase polymerized antibody) for 8 min. Antibody complexes were visualized using DAB (3,3′-diaminobenzidine), detection 2 × for 10 min. Tissues were counterstained with hematoxylin. Slides were removed from the automated stainer, dehydrated and coverslipped. Unless otherwise specified all reagents were obtained from Leica Microsystems. Quantitative imaging was the performed using Visiopharm image analysis software (Visiopharm, Inc., Hoersholm, Denmark). Regions of interest within each tissue were outlined corresponding to the amnion, chorion and decidua within the chorioamniotic membranes and alveoli within the fetal lung. The ratio of the immunostained area of tissue to the area of the entire tissue within the region of interest was calculated and compared across groups using one-way ANOVA with adjustment for multiple comparisons using Tukey's test.

### NET Immunostaining

We performed immunostaning as previously described (75). Briefly, full thickness paraffin sections of fetal roll were deparaffinized and epitopes were revived overnight at 60°C in citrate buffer (10 mM sodium citrate, 0.05% Tween 20, pH 6.0). After epitope revival, slides were washed, blocked for 1 h at 23°C in 5% (v/v) goat serum and 1% (w/v) BSA, then incubated overnight at 4°C with rabbit anti-neutrophil elastase antibody (1:2000, Abcam).Anti-neutrophil elastase antibody was detected using anti-rabbit IgG antibody conjugated to Cy3 (1:2500, Abcam). Extracellular and nuclear DNA was detected using DAPI (4′,6-diamidino-2-phenylindole) in Vectashield (Vector Laboratories). Images were captured using a DM4000B Fluorescent upright microscope (Leica) under 20 × magnifications. The microscope was attached to a DFC310FX camera (Leica) and the acquisition software used was the Leica application suite (version 4.0.0).

### Isolation of Neutrophils From Adult Human Blood

Neutrophils were isolated from fresh human adult blood as described (75). Briefly, ~20–30 mL of blood was collected from independent healthy human donors in EDTA tubes (BD Bioscience). Neutrophils were then isolated using a MACSxpress neutrophil isolation kit following manufacturer's instructions (Miltenyi Biotec). Following neutrophil isolation, the cells were pelleted and any residual red blood cells (RBC) were removed using the RBC lysis solution (0.15 nM NH_4_Cl, 1 mM NaHCO_3_) as per manufacturer's instructions. Cells were then washed with RPMI 1640 containing L-glutamine (Corning Cellgro; hereafter referred to as RPMI-G). Cells were counted and resuspended at appropriate concentrations in assay-specific buffers.

### Neutrophil Killing Assays

Neutrophils isolated as described above were washed in RPMI-G. GBS were grown to early log phase (OD_600_ = 0.3) in TSB (Tryptic Soy Broth) and washed twice with PBS. Approximately 2.2 × 10^4^ neutrophils were incubated with GBSΔ*covR* at an MOI of 10 in RPMI-G in the presence and absence of 100 μg BSCI in nuclease-free water (Ambion Nuclease-Free Water; hereafter referred to as NFW). After 1 h of incubation at 37°C, Triton X-100 was added at a final concentration of 0.025% to release intracellular bacteria, and total bacteria (intracellular and extracellular bacteria) were enumerated by serial dilution and plating. The survival index was calculated as the ratio of CFU recovered in the presence of neutrophils to CFU recovered in the absence of neutrophils. Data shown are representative of two independent experiments with neutrophils isolated from three different donors in each experiment, which was performed in duplicate.

### Neutrophil Reactive Oxygen Species (ROS) Assays

ROS production in neutrophils was measured as described previously (75). Briefly, human neutrophils were isolated as described above and washed in RPMI-G. GBS were grown to early log phase (OD_600_ = 0.3) in TSB and washed twice with PBS. Neutrophils were resuspended in HBSS (Corning) and incubated with 84 μM dihydrorhodamine123 (DHR123) for 30 min at 37°C. Approximately 5 × 10^5^ DHR123-pre-treated neutrophils were incubated with either GBSΔcovR at an MOI of 100 in PBS in the presence and absence of 100 μg BSCI in NFW. Controls included neutrophils (cells only) and neutrophils treated only with BSCI. At various times post incubation (0, 60 min) at 37°C, cells were analyzed on a LSR II flow cytometer to determine oxidation to fluorescent mono-hydrorhodamine 123 (MHR).

### Statistical Analysis

The primary study outcome was PTL, which was hypothesized to be inhibited by pre-treatment with the BSCI. Secondary outcomes were rates of MIAC, fetal sepsis, and adverse pregnancy outcomes (composite of PTL and MIAC/fetal sepsis). Other secondary outcomes included quantities of uterine activity (peak mean daily hourly contraction area), GBS in the amniotic fluid and fetus (CFU), amniotic fluid cytokines and prostaglandins (PGE2, PGF2α), and the myeloperoxidase (MPO; neutrophil marker) and CD68 (macrophage marker) immunohistochemical staining area within the amnion, chorion, decidua and fetal lung. Outcomes were compared using the Fisher's exact test (categorical variables), Wilcoxon rank sum (continuous variables) as appropriate. We pre-specified two comparisons of interest (1) Saline group vs. BSCI and (2) GBS vs. BSCI. We did not adjust our *p*-values *post-hoc* due to the small sample size. Statistical analysis was performed using GraphPad Prism version 8.0 (GraphPad Software, www.graphpad.com) or STATA/IC 14.2 (StataCorp LLC, College Station, TX). Results were considered significant if the *p*-value was ≤ 0.05. However, due to the limited number of samples per group, we also report trends (*p*-values between 0.05 and 0.1) as described previously with NHP experiments (82).

## Results

### BSCI Prevents PTL, but Not MIAC or Fetal Sepsis During GBS Infection

**Figure 1:**
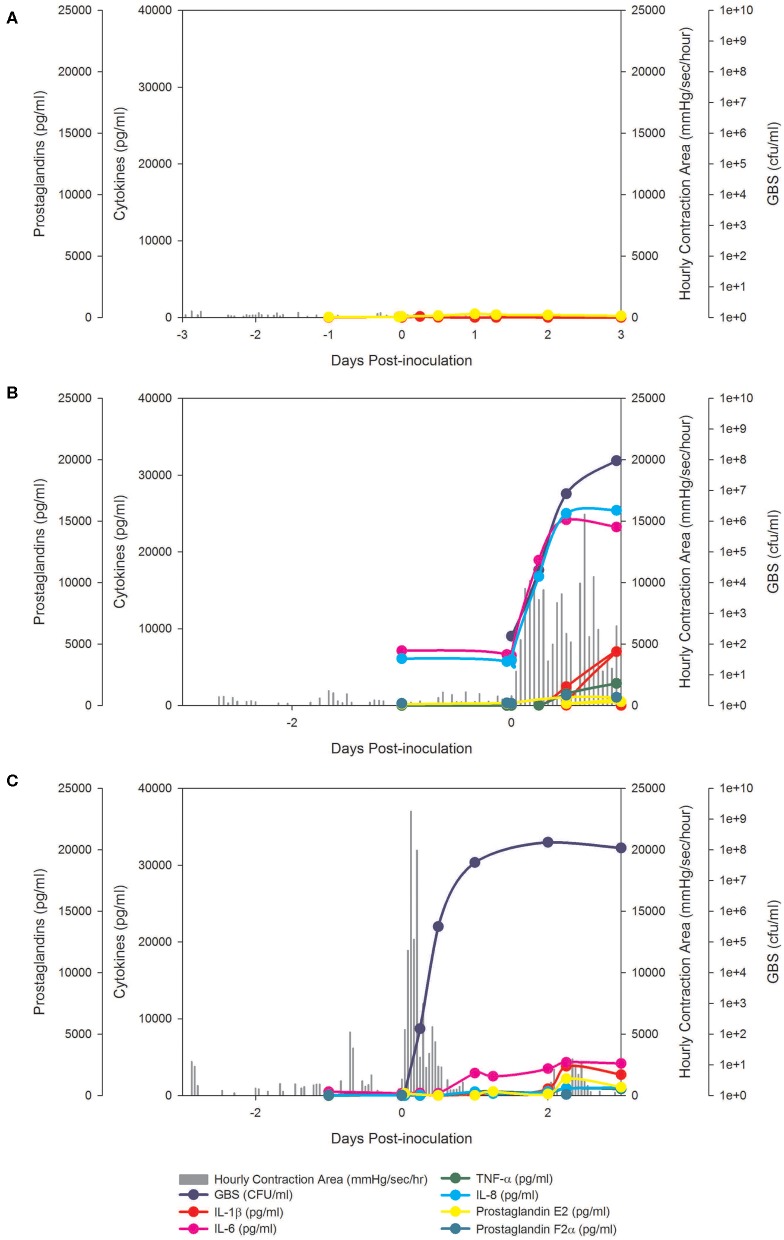
BSCI prevented PTL and inhibited pro-inflammatory cytokines, but did not prevent microbial invasion of the amniotic cavity. In the chronically catheterized NHP model, we obtain intra-amniotic pressure (uterine contraction) continuously and serial samples of amniotic fluid and maternal blood. In these panels, the uterine contraction data (gray bars) and quantities of cytokines, prostaglandins, and GBS are shown. A representative animal is shown that received either a choriodecidual inoculation of saline **(A)**, GBS **(B)**, or GBS with BSCI prophylaxis **(C)**. Typical of other animals in the BSCI+GBS group, GBS inoculation was associated with a transient increase in uterine activity, which was followed by a rapid return to baseline uterine activity (shown in **C**).

We tested whether pre-treatment with a BSCI might suppress GBS-associated PTL and allow for bacterial resolution in a chronically catheterized pregnant NHP model. In our saline controls (*N* = 7), there were no cases of PTL by 7 days post-inoculation (Figure 1A; Table 1). As we previously reported, choriodecidual inoculation of GBS alone induced PTL in 4 of 5 cases (80%) by 3 days post-inoculation (Figure 1B) (75). In contrast, PTL did not occur in any cases inoculated with GBS and treated with the BSCI within this time-frame (0/4, 0%; *p* = 0.02; Figure 1C). Two BSCI animals were delivered 1 day early due to discoloration of the amniotic fluid indicating a significant bacterial burden, which was a pre-defined study endpoint to avoid stillbirth. In one case, clinical microbiological methods to identify bacteria recovered from the amniotic fluid, revealed that contaminating bacteria (*Staphylococcus epidermidis*) was present in one BSCI case prior to GBS inoculation (BSCI case 2, Table 2). We chose to include this case, because it represented a further test of the ability of the BSCI to inhibit microbial-infection associated PTL and biased our study toward the null hypothesis. Notably, despite the presence of at least two different bacterial strains in BSCI case 2, PTL did not occur.

**Table 1:** Summary of pregnancy outcomes, uterine activity, cytokines, and prostaglandins.

**Outcome or cytokine**	**Saline (*N* = 6)**	**GBS (*N* = 5)**	**BSCI+GBS (*N* = 4)**	***P*****-value**	
				**Saline vs. BSCI+GBS**	**GBS vs. BSCI+GBS**
**MATERNAL AND FETAL OUTCOMES**					
PTL	0 (0%)	4 (80%)	0 (0%)	NS	0.02
MIAC	0 (0%)	3 (60%)	4 (100%)	0.005*	NS
Fetal Bacteremia	0 (0%)	3 (60%)	4 (100%)	0.005*	NS
Adverse outcomes^‡^	0 (0%)	5 (100%)	4 (100%)	0.005*	NS
**UTERINE CONTRACTILITY**					
Peak Mean HCA (mm Hg∙s/h)	1,327 (507)	4,008 (763)	3,654 (964)	0.06	NS
Final HCA (mm Hg∙s/h)	607 (434)	3,843 (847)	2,427 (1,189)	0.06	NS
**AMNIOTIC FLUID CYTOKINES AND PROSTAGLANDINS (ng/mL)**					
IL-1β	0.03 (0.02)	5.1 (3.1)	5.8 (2.1)	0.01*	NS
TNF-α	0.03 (0.01)	2.0 (1.2)	1.0 (0.2)	0.01*	NS
IL-6	7.1 (2.6)	22.7 (1.5)	11.8 (7.6)	NS	NS
IL-8	0.9 (0.3)	15.7 (4.2)	3.6 (1.6)	0.06	0.03*
PGE2α	0.5 (0.2)	4.5 (4.1)	3.7 (1.3)	0.03*	NS
PGF2α	0.4 (0.2)	98.5 (98.0)	1.6 (0.8)	NS	NS
**FETAL CYTOKINES (pg/mL)**					
IL-1β	0.9 (0.6)	42.3 (23.2)	3.6 (1.0)	0.02*	0.05
TNF-α	2.3 (1.1)	7.8 (0.8)	3.2 (1.8)	NS	<0.05*
IL-6	3.6 (1.9)	675.3 (413.0)	241.8 (218.0)	0.01*	NS
IL-8	574 (314)	4,721.7 (1745.0)	1,098.4 (885.0)	NS	0.09

*BSCI, broad spectrum chemokine inhibitor; GBS, Group B Streptococcus; HCA, hourly contraction area; IL, interleukin; PTL, preterm labor; MIAC, Microbial invasion of the amniotic cavity; PGE2, prostaglandin E2; PGF2α, prostaglandin F2 alpha; TNF-α, tumor necrosis factor alpha*.

‡ *Adverse outcomes represent PTL and/or MIAC*.

*Maternal and fetal outcomes are shown as number (%) and are compared using Fisher's exact test. Amniotic fluid cytokines and prostaglandins are shown as mean peak (standard error of the mean) in ng/mL. Fetal plasma cytokines (pg/mL) represent the mean quantity at delivery (GBS and BSCI+GBS) or at their peak (saline controls) with standard error of the mean shown in parentheses. Cytokines and prostaglandins are compared between the saline and BSCI+GBS group and the GBS and BSCI+GBS group*.

* *P < 0.05 are considered significant, but p ≤ 0.1 are shown due to the small sample size*.

**Table 2:** Primary outcomes and time course for MIAC and delivery by animal.

**Animal**	**MIAC and fetal sepsis**	Timepoint^*^ when GBS first detected in AF	**PTL**	Days from inoculation^**^ to delivery	**Notes and figure correspondence**
Saline 1	N/A	N/A	N/A	7	
Saline 2	N/A	N/A	N/A	7	
Saline 3	N/A	N/A	N/A	7	
Saline 4	N/A	N/A	N/A	7	Figure 1A
Saline 5	N/A	N/A	N/A	7	
Saline 6	N/A	N/A	N/A	7	
GBS 1	Yes	0.25 h	Yes	0.3 (7 h)	
GBS 2	Yes	0.75 h	Yes	1	Figure 1B
GBS 3	Yes	12 h	No	2	AF brown and cloudy, but no PTL. Delivered due to concern for impending stillbirth.
GBS 4	No	N/A	Yes	3	Lowest GBS inoculum (5 × 10^7^ CFU)
GBS 5	No	N/A	Yes	1	
BSCI+GBS 1	Yes	6 h	No	2	AF brown and cloudy
BSCI+GBS 2	Yes	Non-GBS contaminant detected at −1 h	No	2	AF brown and cloudy; also had a non-GBS contaminant
BSCI+GBS 3	Yes	6 h	No	3	Figure 1C
BSCI+GBS 4	Yes	6 h	No	3	

PTL, preterm labor; MIAC, microbial invasion of the amniotic cavity; AF, amniotic fluid.

* *Indicates the first time point when GBS was cultured from amniotic fluid. For example, a positive value at 6 h indicates that bacterial trafficking occurred sometime between 45 min and 6 h*.

** *The day of inoculation is considered day 0*.

Analysis of the uterine contraction pattern revealed that GBS inoculation alone resulted in a building contraction pattern within 6 h of inoculation that typically culminated in PTL. In contrast, BSCI prophylaxis was associated with a transient increase in uterine activity following GBS inoculation that decreased over time, but did not result in cervical change or PTL. A quantitative analysis of peak mean uterine contraction strength and frequency (hourly contraction area, HCA) revealed that BSCI+GBS and GBS groups had similar peak mean HCA (Table 1); however, at the end of the experiment, uterine contractility was lower in the BSCI+GBS compared to the GBS group (Table 1). Overall, BSCI prophylaxis was associated with inhibition of PTL by 3 days post-GBS inoculation compared to GBS alone.

**Figure 2:**
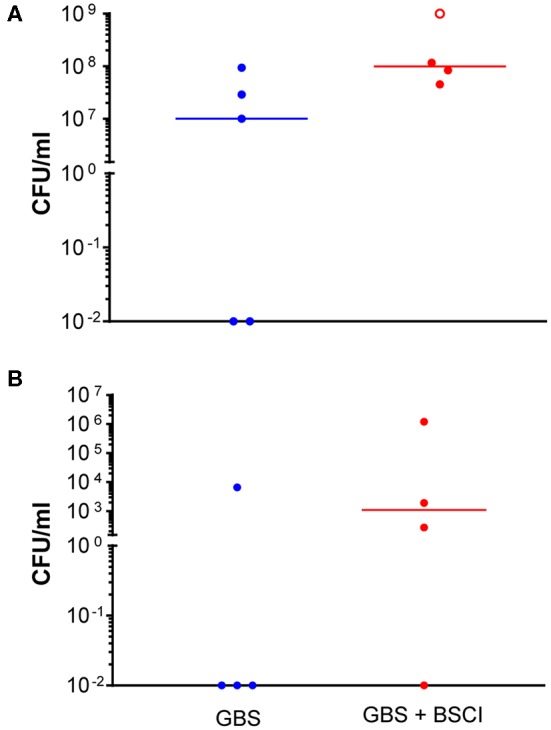
Increased GBS bacterial burden in the amniotic fluid and fetal blood of BSCI-treated animals. GBS CFU are shown in amniotic fluid **(A)** and fetal blood **(B)** obtained at Cesarean section. Animals inoculated with GBS are shown as solid blue circles and those inoculated with GBS but also given BSCI are shown as solid red circles. One animal treated with the BSCI and inoculated with GBS was also found to have a contaminating bacteria (*S. epidermis*) within the amniotic fluid, which complicated estimation of the GBS CFU; this animal is represented by an open red circle.

Next, we sought to determine whether BSCI administration can prevent MIAC and fetal sepsis due to GBS inoculation. Neither MIAC nor fetal sepsis occurred in the saline controls (0/6; 0%). As previously reported, choriodecidual inoculation of GBS alone frequently led to MIAC and fetal bacteremia (3/5, 60%) (75). Notably, in all cases, BSCI administration followed by GBS inoculation was associated with MIAC and/or fetal bacteremia (4/4, 100%). In contrast to our hypothesis, an increased bacterial burden in AF and fetal tissues was noted in the BSCI+GBS group (Figure 2A, 10-fold median increase in AF, *p* = 0.1; Figure 2B, 1,000-fold median increase in fetal blood, *p* = NS) compared to the GBS only group. In all BSCI cases, MIAC occurred by 6 h following choriodecidual GBS inoculation whereas bacteria was detected in the AF by 1 h in 2 GBS cases and in a third GBS case by 12 h post-inoculation (Table 2, Figure 2). Taken together, these data indicate that while administration of BSCI inhibits PTL, it was associated with bacterial dissemination into the amniotic cavity and fetus.

### BSCI Dampens IL-8 in the Maternal Plasma and AF, but Not in Fetal Plasma

**Figure 3:**
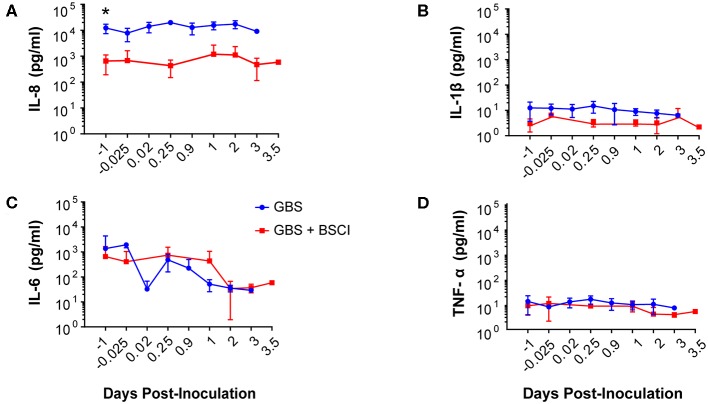
In maternal plasma, BSCI treatment was associated with lower IL-8 **(A)** and IL-1β **(B)** levels at most time points compared to the GBS group, but this was only significant prior to experimental start for IL-8. IL-6 **(C)** and TNF-α levels **(D)** in the maternal plasma of the BSCI and GBS groups were not significantly different by Wilcoxon rank sum. *p < 0.05.

**Figure 4:**
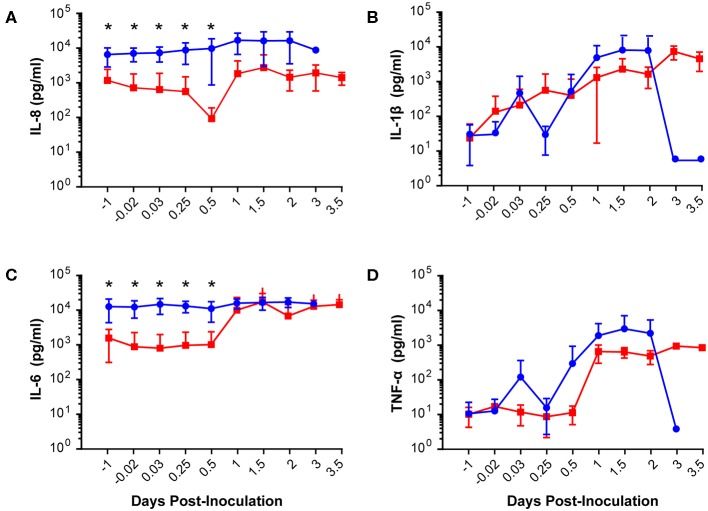
Amniotic fluid quantities of IL-8 **(A)**, IL-1β **(B)**, IL-6 **(C)**, and TNF-α **(D)** are plotted at each time point. The GBS group is shown as solid blue circles and BSCI as solid red circles. Prior to experimental start and for the first 12 h of the experiment, quantities of IL-8 and IL-6 were significantly lower in the BSCI vs. the GBS group by Wilcoxon rank sum. *p < 0.05.

**Figure 5:**
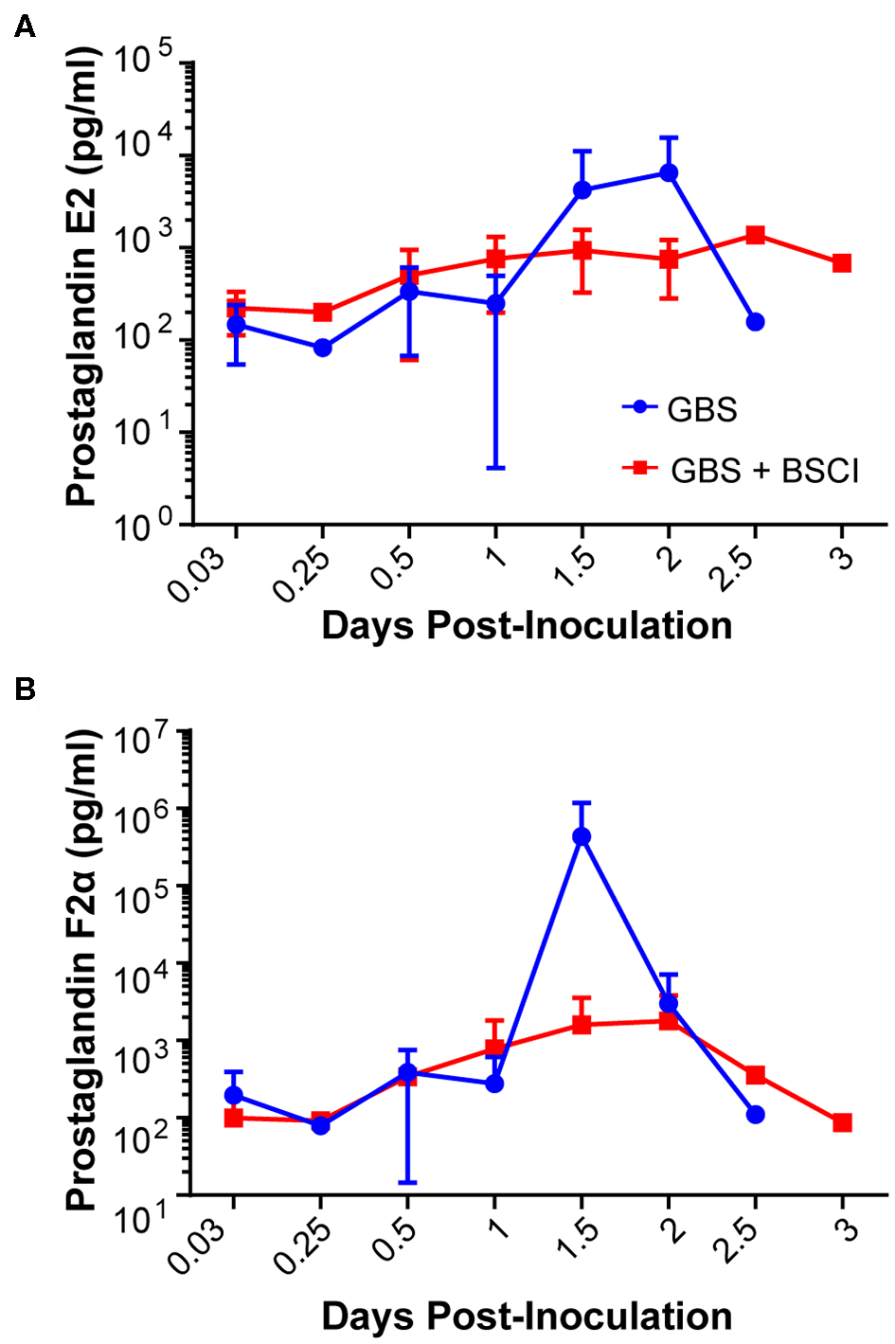
Amniotic fluid quantities of prostaglandin E2 (PGE2, **A**) and prostaglandin F2α (PGF2α, **B**) are shown at each time point for the GBS (blue) and BSCI+GBS group (red). Levels were not significantly different between the BSCI and GBS groups by Wilcoxon rank sum.

Our chronically catheterized NHP model allows for the serial sampling of amniotic fluid and maternal blood throughout the course of the experiment. We therefore examined the effect of BSCI administration on cytokine and chemokine profiles in the maternal blood, amniotic fluid and fetal blood using a luminex assay. BSCI prophylaxis was associated with lower levels of IL-8 in the maternal plasma compared to the GBS group at multiple time points throughout the experiment (Figure 3A). IL-1β levels in maternal plasma were also lower in the BSCI+GBS vs. GBS group (Figures 3B). IL-6 and TNF-α levels in the maternal plasma were not affected by BSCI prophylaxis (Figures 3C,D). In the AF, BSCI+GBS compared to GBS group was associated with significantly lower IL-8 levels for the first 12 h of the experiment (Figure 4A) and significantly lower peak mean levels compared to the GBS group (*p* = 0.03, Table 1); similarly, IL-6 levels were significantly lower for the first 12 h following GBS inoculation (Figure 4C). Levels of AF IL-1β and TNF-α (Figures 4B,D) and AF prostaglandin E2 and F2α (Figure 5) were unaffected by BSCI treatment. In fetal plasma from the BSCI+GBS compared to the GBS group, the peak mean levels of many cytokines were lower including TNF-α (*p* < 0.05), IL-1β (*p* = 0.05), and IL-8 (*p* = 0.09; Table 1). Levels of fetal plasma IL-6 were similar between the GBS and BSCI+GBS groups.

### BSCI Diminished Fetal Chemokines and Cytokines in Tissues but did Not Prevent Bacterial Dissemination

**Figure 6:**
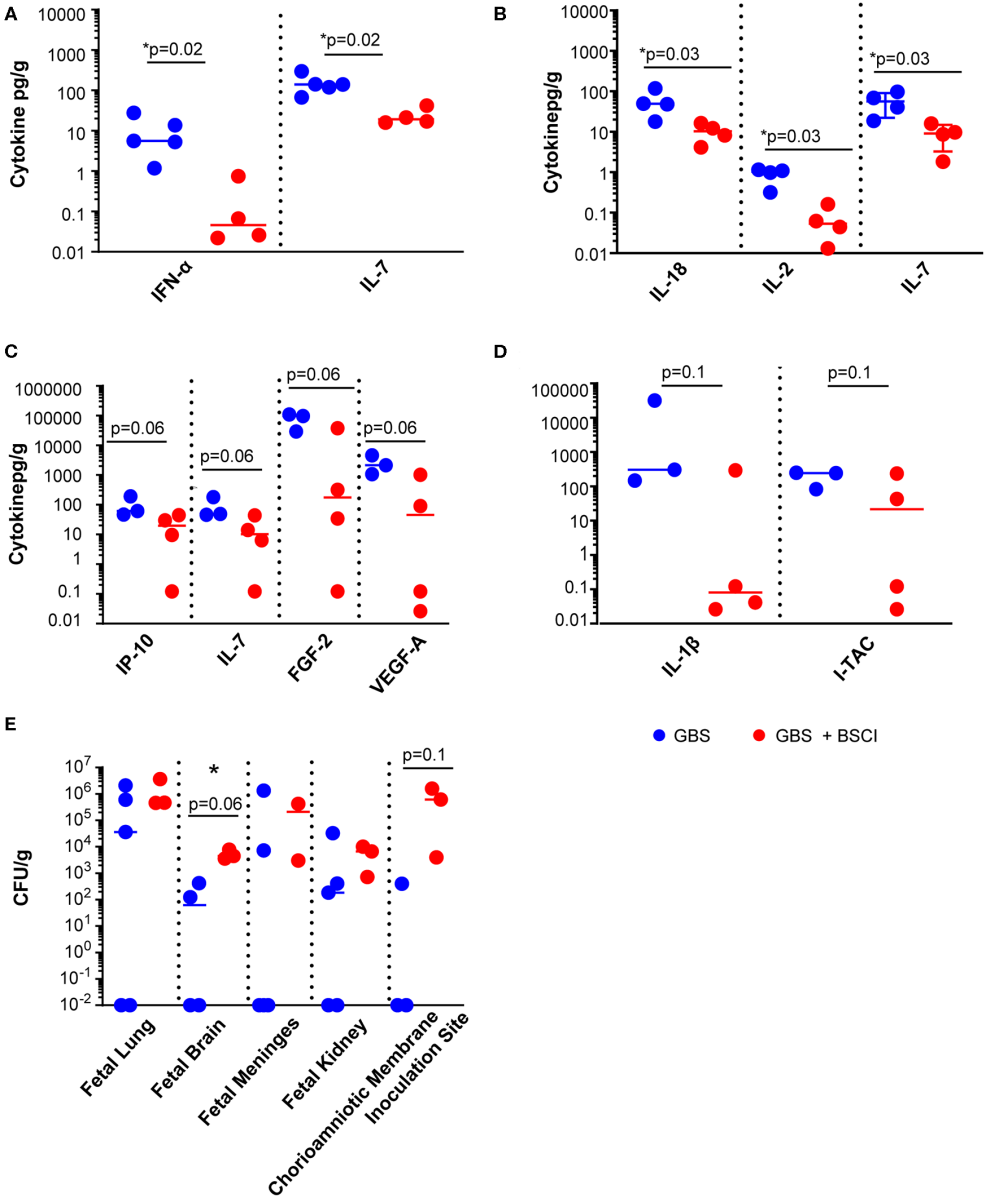
BSCI treatment was associated with a reduction in cytokines and chemokines, but increased CFU counts in fetal tissues. Luminex analysis was performed on **(A)** lung, **(B)** brain, and **(C,D)** chorioamniotic membrane lysates from GBS-infected animals (solid blue circles) and those that received GBS plus BSCI (solid red circles). The CFU counts per gram of tissue **(E)** are shown from GBS-infected animals (solid blue circles) and those inoculated with GBS plus BSCI (solid red circles). *p < 0.05, Wilcoxon rank sum test.

We next asked whether tissue levels of chemokines and cytokines were altered as a result of fetal exposure to the BSCI and focused investigation on the fetal lungs, brain and chorioamniotic membranes at the GBS inoculation site. BSCI prophylaxis was associated with a significantly lower level of IL-7 and IFN-α in the fetal lung (Figure 6A, *p* = 0.02 for both) and significantly lower IL-2, IL-7, and IL-18 in the fetal brain (Figure 6B; *p* = 0.02 for IL-2 and IL-18; *p* = 0.03 for IL-7) compared to GBS alone. Notably, statistical power was limited for these comparisons as we had fewer chorioamniotic membrane inoculation site samples available for Luminex analysis (GBS group, *N* = 3; BSCI+GBS, *N* = 4). The quantities of many other cytokines and growth factors were also lower at the GBS inoculation site within the chorioamniotic membranes with BSCI exposure compared to the GBS only group (Figure 6C; IP-10, IL-7, FGF2, VEGF-A, *p* = 0.06 for all). A trend toward a lower IL-1β and I-TAC concentration at the GBS inoculation site of the chorioamniotic membranes was also observed in the BSCI+GBS group vs. GBS only (Figure 6D, *p* = 0.1).

Diminished levels of pro-inflammatory cytokines may have an adverse effect in controlling bacterial dissemination. We also noted a trend toward a higher bacterial burden in all tissues from BSCI-treated animals compared to GBS alone, particularly in the fetal brain and at the GBS chorioamniotic membrane inoculation site (Figure 6E). These data suggest that treatment with BSCI may dampen inflammatory cytokines at the cost of increased bacterial burden. This may be due partially to decreased neutrophil recruitment at the membrane inoculation site and in fetal tissues. To investigate the impact of BSCI treatment on neutrophil infiltration of the chorioamniotic membranes and fetal tissues, we compared histopathology of the membrane inoculation site and fetal lungs across the treatment groups.

### BSCI Treated Animals Exhibited Increased Neutrophil and Macrophage Recruitment in the Chorioamniotic Membranes and Fetal Lungs

**Figure 7:**
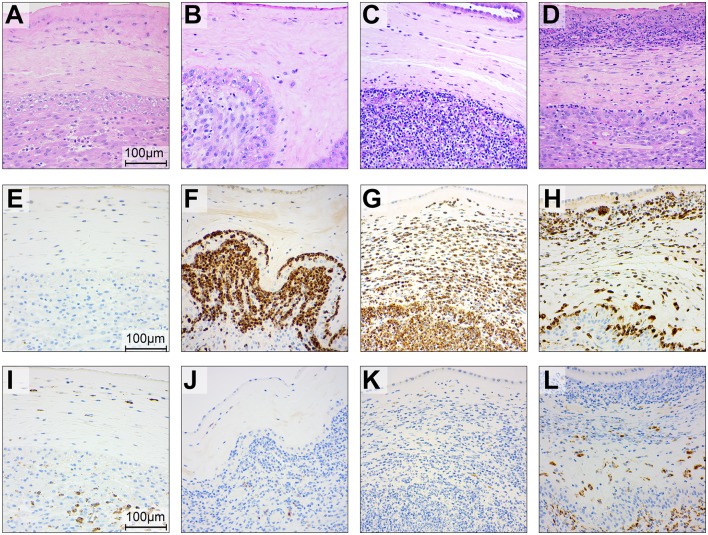
Histopathology and immunohistochemistry of the placental chorioamniotic membranes in saline controls **(A,E,I)**, a GBS case delivered at 24 h **(B,F,J)** and 48 h **(C,G,K)** and with BSCI treatment **(D,H,L)**. Staining for hematoxylin and eosin staining **(A–D)**, MPO **(E–H)**, and CD68 **(I–L)** was performed. In the saline control membranes, there were few macrophages and rare neutrophils **(A)**. GBS inoculation alone was associated with recruitment of neutrophils and a profile that appeared time dependent; neutrophils were typically confined to the decidua by 24 h after GBS inoculation **(B)** and then spread to the chorion and amnion by 48 h **(C)**. Pre-treatment with the BSCI and GBS inoculation was associated with similar features to GBS cases delivered at 48 h, but a greater dispersion of neutrophils throughout the entire chorioamniotic membranes (**D**, 72 h after GBS inoculation). Immunostaining for MPO and CD68 demonstrated a subjective increase in MPO+ **(H)** and CD68+ cells **(L)** in the BSCI treated vs. GBS alone **(F,G,J,K)** and saline **(E,I)** groups.

**Figure 8:**
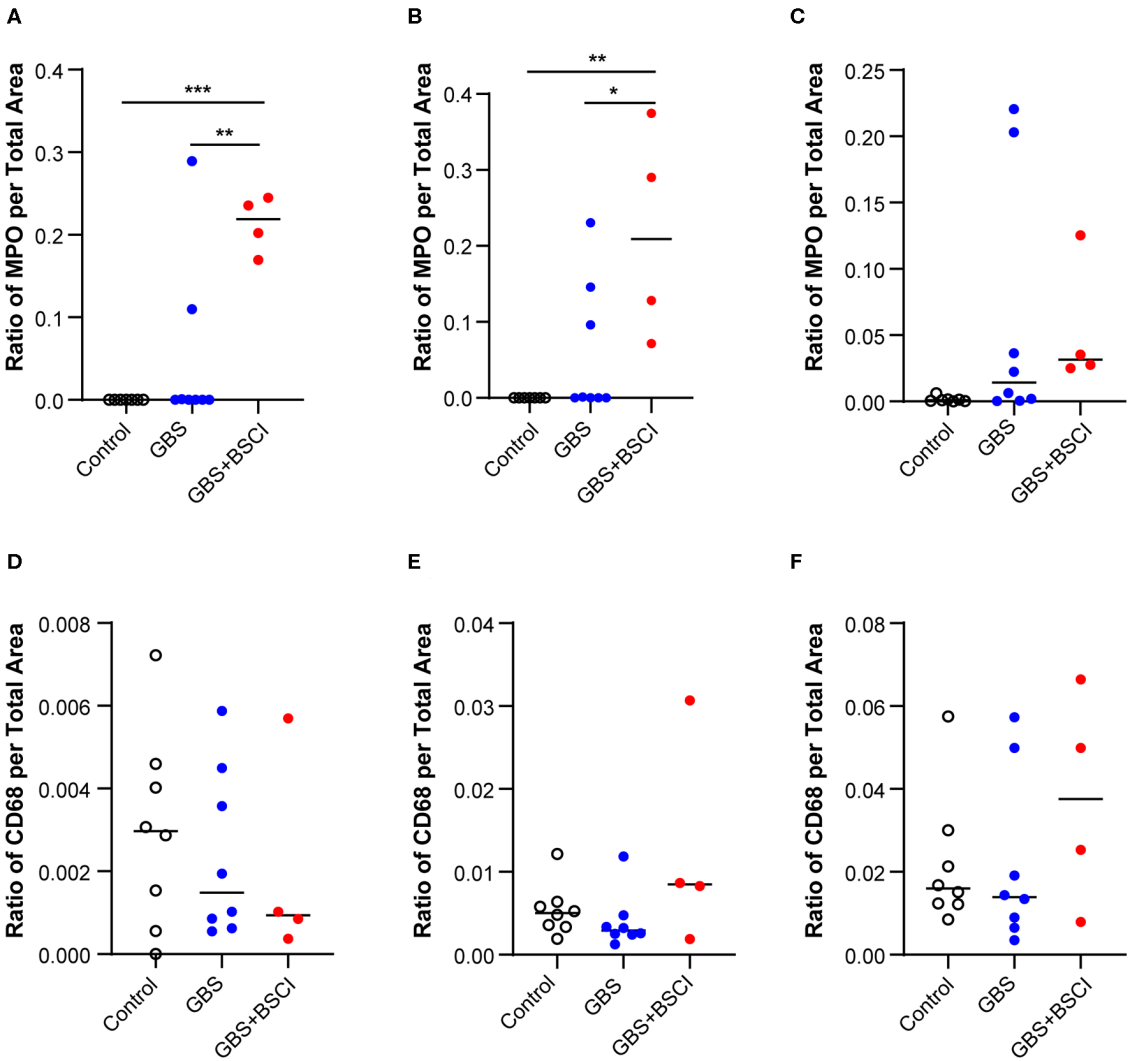
Quantitation of immunostaining for MPO and CD68 in the chorioamniotic membranes. The area of immunostaining for MPO **(A–C)** and CD68 **(D–F)** are shown in the amnion **(A,D)**, chorion **(B,E)**, and decidua **(C,F)**. The area of MPO immunostaining in the BSCI vs. GBS alone group was significantly greater in the amnion **(A)** and chorion **(B)**, but not decidua. No significant differences were observed in the area of CD68 immunostaining between the BSCI and GBS alone groups in the chorioamniotic membranes. The outlier in the saline group in **(D,F)** was a newer saline control performed as part of this study and the next two highest controls were previously published (72). *p < 0.05, **p < 0.01, ***p < 0.001, one-way ANOVA with Tukey's *post-hoc* correction.

To gain further insight into the effect of BSCI on chemokine inhibition, we performed immunostaining on the chorioamniotic membranes at the GBS inoculation site (Figures 7, 8). In the saline controls, there was minimal to no neutrophil infiltration [Figure 7A, H&E stain; Figure 7E, myeloperoxidase (MPO) stain of granulocytes] or CD68 immunostaining (Figure 7I). GBS inoculation led to an accumulation of neutrophils in the decidua at 24 h post-inoculation (Figures 7B,F) and throughout the chorioamniotic membranes by 48 h (Figures 7C,G). Albeit unexpected, we observed that inhibition of chemokines by BSCI did not significantly dampen neutrophil migration into the chorioamnion. Rather, increased neutrophil migration was observed in the BSCI+GBS group when compared to the GBS group (Figures 7D,H), which may in part reflect the longer latency until delivery in the BSCI+GBS group as these animals did not develop PTL. Quantitation of the immunostained regions of the membranes revealed a significantly higher area of MPO+ stained cells in the amnion and chorion, but not the decidua, in the BSCI+GBS compared to the GBS only group (Figures 8A–C). CD68 immunostaining was similar across groups in the amnion, chorion and decidua (Figures 8D–F); although CD68 immunostaining in the decidua appeared greater in the BSCI vs. GBS and saline groups (Figures 7I–L), this was not significant and driven mainly by staining in 2 of the 4 BSCI cases (Figure 8F). These results suggest that although BSCI dampened the levels of certain chemokines, this did not impact neutrophil recruitment into the membranes. Despite the increase in neutrophil recruitment, BSCI was also unable to limit bacterial invasion of the amniotic cavity and fetal organs.

**Figure 9:**
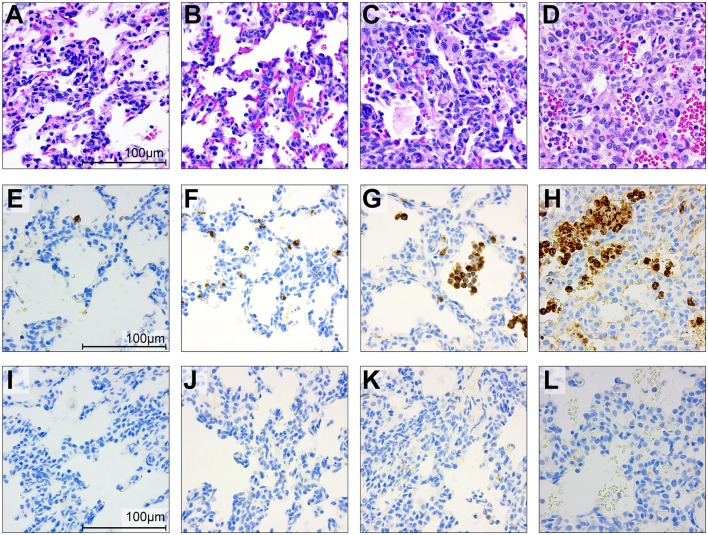
Histopathology and immunohistochemistry of the fetal lungs in saline controls **(A,E,I)**, a GBS case delivered at 24 h **(B,F,J)** and 48 h **(C,G,K)** and with BSCI treatment **(D,H,L)**. Staining for hematoxylin and eosin staining **(A–D)**, MPO **(E–H)**, and CD68 **(I–L)** was performed. In the fetal control lungs, there were few macrophages and rare neutrophils **(A)**. GBS inoculation alone was associated with recruitment of neutrophils and macrophages, congestion and alveolar hemorrhage. Pre-treatment with the BSCI and GBS inoculation was associated with similar features, but greater neutrophil recruitment and more severe histopathologic features of pneumonia (**D**, 72 h after GBS inoculation). Immunostaining for MPO and CD68 demonstrated a subjective increase in MPO+ cells **(H)** in the BSCI treated vs. GBS alone group **(F,G,J,K)** at 72 h post-inoculation.

**Figure 10:**
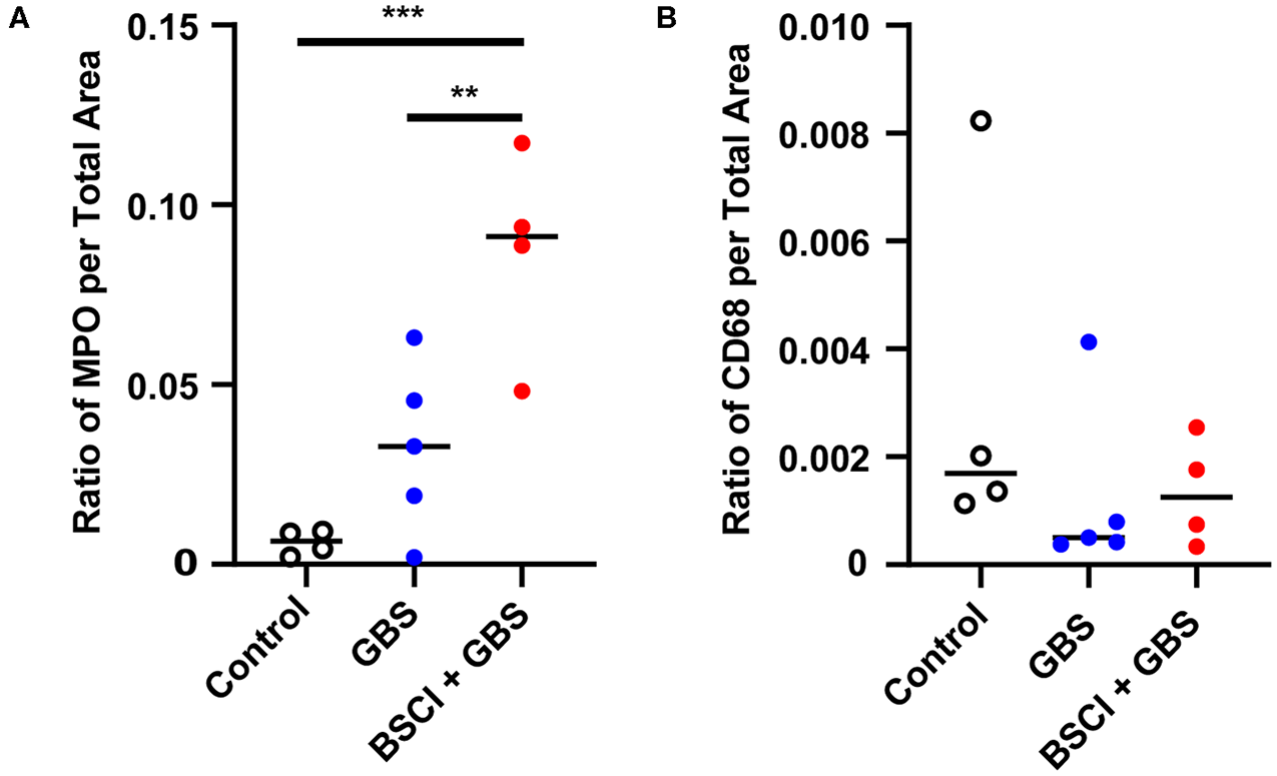
Quantitation of immunostaining for MPO and CD68 in the fetal lung. The area of immunostaining for MPO **(A)** and CD68 **(B)** is shown within the fetal lung parenchyma as quantitated using Visiopharm software. The MPO immunostaining area was significantly greater in the BSCI treatment group compared to GBS alone or saline controls. No significant difference was observed in the CD68 immunostaining area between the BSCI and other groups in the fetal lung. The outlier in the saline group in panel B was a newer saline control performed as part of this study. **p < 0.01, ***p < 0.001, one-way ANOVA with Tukey's *post-hoc* correction.

We also investigated the effect of BSCI exposure on the fetal lungs (Figures 9A–D), which are in direct contact with the amniotic fluid, a site of BSCI inoculation. Recruitment of neutrophils and CD68+ cells into the fetal lungs followed a similar pattern as in the chorioamniotic membranes with a significantly increased immunostaining area of MPO+ cells in the BSCI+GBS group (Figures 9H,10A) vs. GBS alone (Figures 9F,G, 10A) or saline (Figures 9E, 10A). There was no change in the area of CD68 immunostaining in the BSCI compared to either the GBS or saline groups (Figures 9I–L, **10B**). Notably, the GBS bacterial burden in the fetal lungs was similar or higher in the BSCI+GBS group compared to the GBS group (Figure 6E). Therefore, increased neutrophil recruitment was not effective in reducing the GBS burden within the fetal lung at 3 days post-inoculation suggesting either ineffective bacterial killing or insufficient time for neutrophils to clear bacteria by the study endpoint.

### BSCI did Not Impact Neutrophil Function

**Figure 11:**
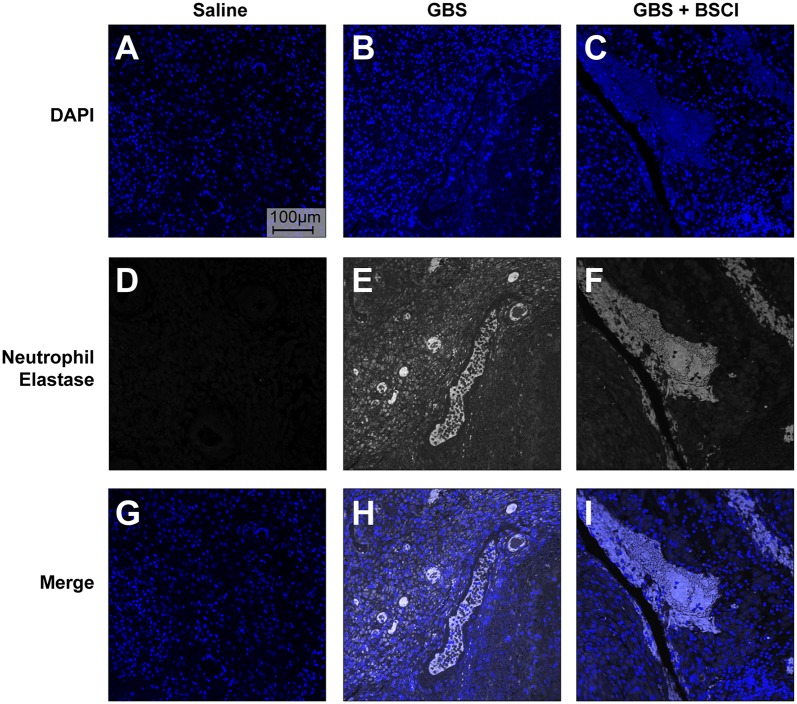
BSCI does not impact neutrophil extracellular traps formed in the chorioamnion during GBS infection in our NHP model. Immunofluorescence staining for neutrophil elastase was performed on chorioamniotic membranes in the saline **(A,D,G)**, GBS **(B,E,H)**, and the BSCI+GBS group **(C,F,I)**. Data shown is representative of five animals from each group. Neutrophil elastase staining is shown in gray and DAPI is shown in blue.

Our previous studies indicate that the GBS strain, used in this study, induced the formation of neutrophil extracelluar traps (NET) (75). Extracellular release of neutrophil contents such as neutrophil elastase and DNA has been associated with formation of NETS and bacterial entrapment (83, 84). Co-localization of neutrophil elastase and DNA in the chorioamnion of GBS infected animals previously revealed the presence of NETs (75). Therefore, we examined whether BSCI impacted formation of NETs induced by choriodecidual GBS infection (Figure 11). We used human adult neutrophils as a model for how BSCI might impact maternal and fetal neutrophils. Immunofluorescence staining for neutrophil elastase indicates that while NET formation is seen in the GBS infected animals, there was no difference in NET staining density in the chorioamniotic membranes of the BSCI+GBS and GBS only groups (Figure 11).

**Figure 12:**
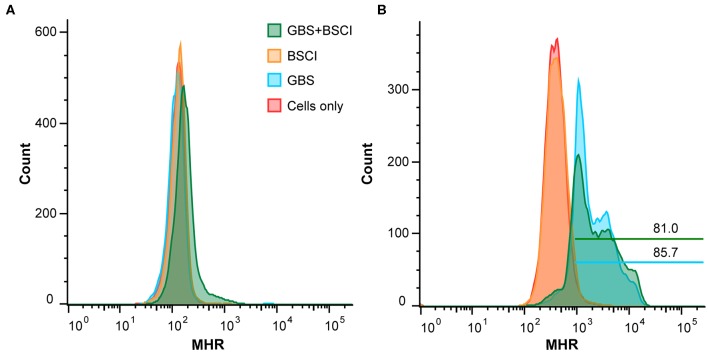
BSCI does not impact neutrophil production of ROS or survival. Adult human neutrophils were pretreated with 84 μM di-hydro-rhodamine 123. Subsequently, the neutrophils were treated with GBS in the presence or absence of BSCI and oxidation to fluorescent mono-hydrorhodamine 123 was monitored at baseline **(A)** or 60 min after GBS and/or BSCI treatment **(B)**. Gates shown reflect the percentage of all cells. Adult human neutrophils were incubated with GBS in the presence or absence of BSCI. The total surviving bacteria (intracellular and extracellular) were enumerated 1 h after incubation. The survival index was calculated as the ratio of colony forming units (CFU) recovered in the presence of neutrophils to CFU recovered in the absence of neutrophils. Data shown are the average of two independent experiments with neutrophils isolated from three different donors for each experiment, which was performed in duplicate, error bars ± SEM.

We also tested whether neutrophil function was impaired in the context of BSCI treatment. To measure production of reactive oxygen species (ROS), human adult neutrophils were pre-treated with di-hydro-rhodamine 123 (DHR) before exposure to GBS. In the presence of ROS, DHR is oxidized to mono-hydro-rhodamine (MHR) which can be quantitated using flow cytometry. The number of ROS positive cells was not significantly different between neutrophils exposed to GBS vs. neutrophils exposed to GBS and BSCI (Figures 12A,B). Finally, GBS survival in the presence of adult human neutrophils was not significantly different as a result of BSCI exposure (Figure 12C). Together, these data suggest that the increased bacterial burden observed in the BSCI treated animals was not due to a BSCI-mediated suppression of neutrophil function or bacterial killing, but may reflect a generalized impairment of the innate immune response and/or insufficient time to clear a rapidly progressing bacterial infection.

## Discussion

In this study, our primary objective was to determine if the BSCI could inhibit PTL and whether there was a beneficial or detrimental effect on bacterial trafficking in the amniotic cavity. Our results demonstrate that administration of a BSCI during an invasive microbial infection, led to the powerful suppression of uterine activity and a complete blockade of PTL at 3 days post-inoculation. Although the inhibition of PTL was remarkable, BSCI did not prevent bacterial trafficking to the amniotic cavity and fetal infection. The simultaneous progression of infection into the fetal compartment represents a serious adverse outcome for the pregnancy. Fetal bacteremia occurred in all of the experiments involving BSCI treatment, compared to only 20% of animals receiving GBS alone. In the BSCI+GBS group, there was a significant reduction in IL-8 in the maternal blood and amniotic fluid compared to the GBS only group; many other cytokines (e.g., IL-6, IL-1β, IL-7) were reduced in either the fetal plasma, lung, or brain compared to GBS alone. Although one might expect a reduction in neutrophilic infiltration due to the BSCI, there was a significantly greater quantity of neutrophils in the chorioamniotic membranes and fetal lungs of the BSCI animals compared to the GBS only group. Overall, the lack of uterine contractile activity in the BSCI+GBS group was remarkable, particularly in the context of the significant infectious burden in the chorioamniotic membranes, amniotic fluid, and fetal compartment.

BSCIs are an attractive therapeutic class of drugs for the inhibition of PTL, because they have the capability to simultaneously block multiple cytokine signaling pathways (51, 55). BSCIs are unlike other anti-inflammatory drugs in that they do not antagonize the classical pathway of chemokines binding to their receptors, but rather they act as agonists by binding to cell-surface type-2 somatostatin receptors (SSTR2). A total of five sub-types of human SSTR have been cloned and detected in human tissues with splice variants SSTR2A and SSTR2B (85, 86). SSTRs (mainly SSTR2 and SSTR5) are reported to be highly expressed on inflammatory cells, including activated lymphocytes, monocytes, and endothelial cells (52, 87). Interestingly, it was shown that binding SSTR2 inhibits adenylyl cyclase and calcium entry by suppressing voltage-dependent calcium channels, which can affect several organ-specific functions (i.e., inhibiting secretion of glucagon and insulin in the pancreas, inhibiting neurotransmitter in the brain) (88–91). BSCIs are partial agonists of SSTR2, which stimulate signals that diminish pathways generated by the related chemokine receptors; without classical SSTR2 agonist effects (e.g., effect on growth hormone levels or the hypothalamic-pituitary axis) (53, 54). Partial agonism of SSTR2 has been hypothesized to dampen directional signals from the chemokine receptors leaving cells effectively “blinded” to the chemokine gradient.

**Figure 13:**
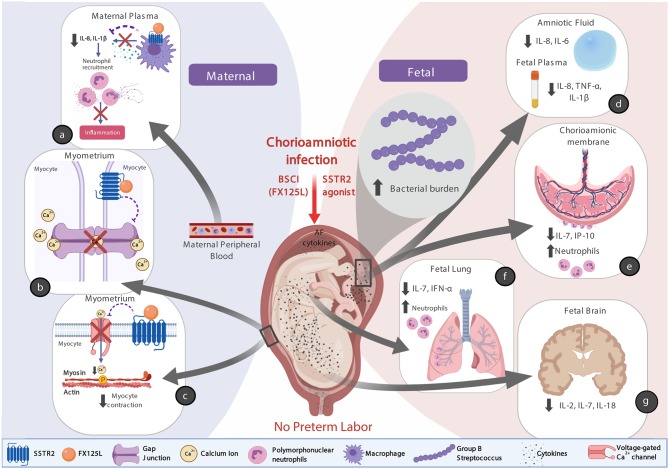
A conceptual model showing how BSCI (FX125L) can prevent PTL, but allow for neutrophilic infiltration into placental chorioamniotic membranes and fetal lungs. We show the maternal **(a)** and fetal **(d–g)** effects of BSCI, as well as proposed mechanisms of BSCI action on the myometrium **(b,c)**. In the BSCI +GBS group compared to the GBS only group, the major neutrophil chemokine IL-8 and pro-inflammatory cytokine IL-1β were reduced in the maternal plasma **(a)** and pro-inflammatory cytokines were inhibited in amniotic fluid and the fetal plasma **(d)**, chorioamniotic membranes **(e)**, fetal lungs **(f)**, and fetal brain **(g)** tissues compared to GBS alone. BSCI administration did not prevent GBS-associated neutrophil recruitment into the fetal membranes and fetal lung **(e,f)**. The simultaneous progression of infection into the fetal compartment represents a serious adverse outcome for the pregnancy. Note that this figure was created with Biorender.com.

There are at least three possible explanations for why the BSCI treatment was so effective in preventing preterm labor (Figure 13, conceptual model). First, we speculate that binding of BSCI to SSTR2 could have inhibited calcium entry by suppressing voltage-dependent calcium channels, which prevented the increase in intracellular calcium ([Ca^2+^]_i_) and thus precludes elevated [Ca^2+^]_i_ to act on the contractile filaments of myometrial smooth muscle cells, and generate uterine contractions. Secondly, it is plausible that the BSCI may have inhibited the expression or function of gap junction proteins, in particular Connexin 43. Propagation of an action potential can only be generated when individual cells are electrically coupled to allow elevated Ca^2+^ to spread between neighboring cells. In the myometrium, this coupling occurs through intercellular bridges formed by gap junction proteins (connexins) through which ions and certain small molecules pass. Connexin 43 is the most abundant connexin in the uterus and its expression is increased with term and preterm labor in all mammals (92). Finally, there was also a significant reduction in the major neutrophil chemokine IL-8 in the maternal plasma and amniotic fluid in the BSCI compared to the GBS only group; many other cytokines (e.g., IL-6, IL-1β, IL-7, TNF-α) were also reduced in either the fetal plasma, chorioamniotic membranes, lung, or brain tissues compared to GBS alone. As IL-8 infusion *alone* does not induce PTL in a pregnant rhesus macaque model (93), this suggests that a broader suppression of chemokines may be a promising approach to inhibiting PTL if microbial infections are properly diagnosed and treated. Altogether, these factors acting individually or in concert may explain why the BSCI+GBS group did not develop PTL.

Interestingly, the cytokine pattern observed in the NHP model is remarkably different from what was observed in our murine model of LPS-induced preterm birth (55). In the NHP model, IL-7 was inhibited in the chorioamniotic membranes, as well as in fetal brain and fetal lungs. IL-7 is a hematopoietic growth factor, produced and secreted by neurons, epithelial, and other cells and is crucial for the maturation of lymphocytes. Further, pro-inflammatory cytokines IL-2 and IL-18 (both capable of regulating adaptive and innate immunity) were also inhibited in the fetal brain. In contrast to the NHP model with many cytokines in the AF and fetal tissues showing decreased levels, only maternal tissues exhibited diminished cytokines in response to BSCI treatment in the murine systemic LPS-induced model of preterm birth (55). This may be due in part to the fact that the BSCI was given to mice intraperitoneally, but not intra-amniotically and intravenously similar to the NHP model. In our previous study, the BSCI (BN83470) compound needed to cross two layers of syncytiotrophoblast cells (mouse placental barrier), which is unlikely due to the chemical structure of the BSCI. Therefore, we speculated that BN83470 was not able to cross the placenta, as we could not detect any effect of this drug on the chemokine profile in the amniotic fluid of mice systemically challenged with LPS. However, in the current study, we administered FX125L both intra-amniotically and intravenously with effects detected within fetal tissues. These data also highlight the many differences between the two models of PTL with the NHP model more closely resembling human pregnancy.

Given our observations that the BSCI treated animals exhibited increased bacterial burden, it is possible that diminished chemotaxis and recruitment of leukocytes early during infection, might inadvertently allow for greater bacterial growth and burden. Alternatively, it is possible that the greater time achieved *in utero* due to BSCI administration and suppression of PTL allowed for a natural progression of infection that would have occurred normally in the GBS only group if given sufficient time; however, PTL in the GBS only group resulted in early delivery in nearly all cases, which truncated the experimental time course. It is also possible that a combination of greater time *in utero* and delayed or blunted leukocyte action contributed to the microbial invasion of the amniotic cavity seen in every animal in the BSCI+GBS group. Interestingly, the striking neutrophilic infiltration of the chorioamniotic membranes and fetal lungs in the BSCI+GBS group suggests that the BSCI despite blunting some inflammatory responses, did not ultimately prevent neutrophil recruitment; further, the increased neutrophil infiltration was not sufficient to contain bacterial growth or induction PTL. Our *in vitro* data indicate that the BSCI did not impact the microbicidal function of neutrophils. Therefore, it is likely that the suppression of certain chemokines by BSCI while delaying PTL promoted increased bacterial replication in the animals inoculated with GBS and treated with the BSCI.

Whether a combination of BSCI and antibiotics might inhibit PTL and eradicate the infection is unknown, but represents an important question and could be an approach to translate to human pregnancy. A second and critical question arising from our results is the precise mechanism of PTL inhibition, which would best be studied by analyzing inflammatory and immune responses regionally within the myometrium and placental tissues. It is notable that suppression of IL-8 was a recurring finding in our cytokine analyses within the amniotic fluid and maternal plasma placental, which represents an attractive focal point for further experiments to define the expression of genes promoting parturition. Future studies to analyze the expression of genes promoting labor within the placenta and myometrial tissues, in particular, may address why PTL was absent following BSCI treatment despite a significant infection.

The strength of our study is in the use of a unique chronically catheterized NHP model, which represents the closest animal model to human pregnancy. Humans and NHP share a number of features in pregnancy, which enables a direct comparison. These experiments in an NHP model afford a rare opportunity to determine how a PTL therapeutic can affect maternal-fetal immune responses, as well as the health of the fetus. Further, this model allows for serial analyses of maternal blood, amniotic fluid, and uterine contraction data over time, which is not possible in lower mammalian models. Our study was limited by the small number of animals within each group, which is typical of NHP studies where conservation is necessary for ethical reasons and experimental costs are typically 1,000–2,000-fold> that of murine studies. This precluded us from studying the direct effect of BSCI on pregnant animals in the absence of infection.

Important questions remain as to whether a therapeutic for PTL can safely inhibit labor without jeopardizing the health of the fetus. Our results strongly suggest that inhibition of PTL using a BSCI may be effective, but it is imperative for the obstetrician to rule out intra-amniotic infection for the safety of the fetus and the mother. Several new studies suggest that an intra-amniotic infection, in some cases, can be treated successfully with antibiotics (94–98). Further research is needed to determine if antibiotic therapy with an immunomodulator, similar to the BSCI, can eradicate an intrauterine infection and prevent PTL. As immunomodulators to prevent preterm birth move from the laboratory into clinical trials, it will be critical that intra-amniotic infections are properly diagnosed and treated with antibiotics to prevent progression of the infection.

## Data Availability Statement

The datasets generated for this study are available on request to the corresponding author.

## Ethics Statement

The studies involving human participants were reviewed and approved by Seattle Children's Research Institute Institutional Review Board (protocol #11117). The patients/participants provided their written informed consent to participate in this study. The animal study was reviewed and approved by the University of Washington Institutional Animal Care and Use Committee.

## Author Contributions

MC, LR, and KA drafted the initial manuscript. MC, NK, AB-R, OS, SL, LR, and KA critically edited the manuscript. MC, AO, T-YW, MD, SM, JO, AB, JM, LR, and KA provided technical assistance with the experiments. The funding was obtained by OS, SL, and KA.

## Conflict of Interest

The authors declare that the research was conducted in the absence of any commercial or financial relationships that could be construed as a potential conflict of interest.
